# Early treatment with minocycline following stroke in rats improves functional recovery and differentially modifies responses of peri-infarct microglia and astrocytes

**DOI:** 10.1186/s12974-018-1379-y

**Published:** 2019-01-09

**Authors:** Wai Ping Yew, Natalia D. Djukic, Jaya S. P. Jayaseelan, Frederick R. Walker, Karl A. A. Roos, Timothy K. Chataway, Hakan Muyderman, Neil R. Sims

**Affiliations:** 1 Centre for Neuroscience, College of Medicine and Public Health, Flinders University, GPO Box 2100, Adelaide, SA 5001 Australia; 2 Hunter Medical Research Institute; School of Biomedical Medical Sciences and Pharmacy, University of Newcastle Priority Research Centre in Stroke and Traumatic Brain Injury, Newcastle, NSW Australia

**Keywords:** Stroke, Focal ischemia, Microglia, Astrocytes, Minocycline, Functional recovery, Peri-infarct, Photothrombosis

## Abstract

**Background:**

Altered neuronal connectivity in peri-infarct tissue is an important contributor to both the spontaneous recovery of neurological function that commonly develops after stroke and improvements in recovery that have been induced by experimental treatments in animal models. Microglia and astrocytes are primary determinants of the environment in peri-infarct tissue and hence strongly influence the potential for neuronal plasticity. However, the specific roles of these cells and the timing of critical changes in their function are not well understood. Minocycline can protect against ischemic damage and promote recovery. These effects are usually attributed, at least partially, to the ability of this drug to suppress microglial activation. This study tested the ability of minocycline treatment early after stroke to modify reactive responses in microglia and astrocytes and improve recovery.

**Methods:**

Stroke was induced by photothrombosis in the forelimb sensorimotor cortex of Sprague-Dawley rats. Minocycline was administered for 2 days after stroke induction and the effects on forelimb function assessed up to 28 days. The responses of peri-infarct Iba1-positive cells and astrocytes were evaluated using immunohistochemistry and Western blots.

**Results:**

Initial characterization showed that the numbers of Iba1-positive microglia and macrophages decreased in peri-infarct tissue at 24 h then increased markedly over the next few days. Morphological changes characteristic of activation were readily apparent by 3 h and increased by 24 h. Minocycline treatment improved the rate of recovery of motor function as measured by a forelimb placing test but did not alter infarct volume. At 3 days, there were only minor effects on core features of peri-infarct microglial reactivity including the morphological changes and increased density of Iba1-positive cells. The treatment caused a decrease of 57% in the small subpopulation of cells that expressed CD68, a marker of phagocytosis. At 7 days, the expression of glial fibrillary acidic protein and vimentin was markedly increased by minocycline treatment, indicating enhanced reactive astrogliosis.

**Conclusions:**

Early post-stroke treatment with minocycline improved recovery but had little effect on key features of microglial activation. Both the decrease in CD68-positive cells and the increased activation of astrogliosis could influence neuronal plasticity and contribute to the improved recovery.

**Electronic supplementary material:**

The online version of this article (10.1186/s12974-018-1379-y) contains supplementary material, which is available to authorized users.

## Background

Spontaneous improvements in neurological function develop progressively over the first few months following stroke. Although multiple changes in the central nervous system contribute to these improvements, alterations in neuronal connectivity in tissue surrounding the infarct are an important component [1–4]. Interventions that have been found to improve neurological recovery after stroke are often associated with additional increases in peri-infarct neuronal plasticity that contribute to the better outcomes [3–6]. Thus, there is considerable interest in understanding factors that can modulate these neuronal responses and promote beneficial changes in connectivity in peri-infarct tissue.

Glial cells are major determinants of the peri-infarct environment and a key influence on the capacity for change in neuronal connectivity. The death of cells in the developing infarct activates microglia in the surrounding viable tissue producing marked changes in morphology and gene expression that are initiated within the first 24 h after stroke [7–11]. Changes in astrocytic properties become prominent over the first few days, triggered in part by substances released from the microglia including cytokines and chemokines [12–16]. Astrocytes are centrally involved in the development of a glial scar that limits the spread of damage but can also impede changes in neuronal outgrowth and connectivity [14, 15]. Astrocytes and microglia also release many other molecules that can influence recovery [11, 12, 15, 16]. The complexity of the response is further enhanced by the migration of blood-borne cells into the infarct and surrounding tissue [17–19]. These include monocytes that become tissue macrophages and share many properties with resident microglia.

Manipulations of glial cells in the peri-infarct tissue have the potential to improve or impair functional recovery. The outcomes of such interventions are likely to be greatly influenced by the timing of the treatments and the specific cellular responses that are targeted. In the present study, we aimed to investigate the consequences of early modification of peri-infarct microglial activation by treating with the anti-inflammatory agent, minocycline, during the first 2 days after stroke. Minocycline is a second-generation tetracycline that has previously been shown to reduce tissue infarction following focal ischemia [20–24]. Moderate improvements in neurological recovery have also been seen with minocycline treatment protocols that do not significantly modify infarct volume [25–27]. Although, minocycline has been reported to influence multiple cellular properties, an ability to impede activation of microglia has been identified as a prominent feature of the actions of this drug in models of stroke and other brain diseases in animals [20, 28]. Thus, we predicted that modifications to peri-infarct microglial activation would be a major component of the cellular responses to early post-stroke treatment with minocycline. This prediction was directly evaluated based on measures derived from immunolabeling of brain sections (as discussed further below). The effects of minocycline treatment on the subsequent development of reactive astrogliosis and the recovery of neurological function were also investigated.

Infarction in the cerebral cortex was produced by photothrombosis within the forepaw sensorimotor cortex in rats [29, 30]. This model of stroke, which results in permanent ischemia, typically generates more reproducible infarcts than other approaches. The infarcts can be generated to occupy a relatively small proportion of the total cerebral cortex, mimicking the situation for those strokes in humans that have a better prospect for recovery [2, 31]. The photothrombotic model has been widely used to identify changes in neuronal connectivity associated with spontaneous improvements in function following stroke and to investigate responses to treatments [2, 5]. The pattern of changes in astrocytes and microglia is similar to that in other models of stroke [32–35]. However, the infarct develops more rapidly in the photothrombotic model. Thus, it is well suited to investigating the effects of treatments on functional recovery without the complication of changes in infarct volume.

Previous investigations of the effects of minocycline on microglia have usually assessed activation of these cells based on qualitative observations of morphology and cell density in brain sections immunolabeled to detect either ionized calcium binding adaptor molecule 1 (Iba1) or CD11b. To better characterize the patterns of activation of peri-infarct microglia in photothrombotic stroke and the consequences of minocycline treatment, we measured two aspects of Iba1 immunolabeling: (i) the “circularity” of labeled cells, which is increased as a result of the morphological changes associated with activation; (ii) the percentage of total area occupied by Iba1 immunolabeling (subsequently described as “area fraction”), which in the peri-infarct tissue predominantly detects alterations in microglial numbers arising from the balance between cell migration, death, and proliferation. These parameters reflect key components of microglial activation that require complex changes in protein function and gene expression in these cells. As with almost all markers for widely expressed microglial proteins, antibodies against Iba1 also label macrophages derived from circulating monocytes that invade the injured brain tissue. Brain sections were co-immunolabeled for the neuronal marker, NeuN, which clearly delineated the infarct at all time points studied, thus facilitating the identification of regions of interest for the analysis.

## Methods

### Experimental design

Male Sprague-Dawley rats were obtained from Laboratory Animal Services (University of Adelaide, Adelaide, Australia). The animals were kept in a temperature-controlled and humidity-controlled room with a 12 h/12 h light/dark cycle and ad libitum access to water and standard rat chow. Stroke was induced in rats weighing between 270 and 340 g.

**Fig. 1:**
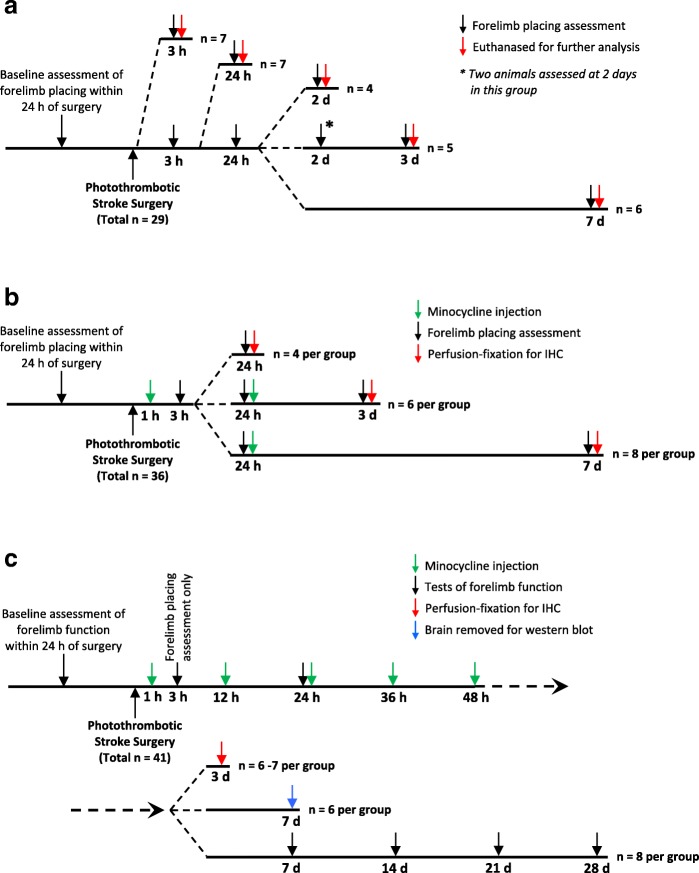
Summary of experimental design for the three major phases of the study. **a** Characterization of responses of Iba1-positive cells in peri-infarct tissue during the first week following photothrombotic stroke. The numbers of rats shown were all assessed using the forelimb placing test. Perfusion-fixed tissue from four to six rats per time point was used to assess infarct volume and that from four rats per time point was used for immunohistochemistry. **b** Effects of lower-dose minocycline treatment on forelimb function and cellular changes during the first week following photothrombotic stroke. **c** Effects of higher-dose minocycline treatment on forelimb function (up to 28 days) and cellular changes following photothrombotic stroke

The study involved three major investigations: A. Characterization of responses of Iba1-positive cells in peri-infarct tissue during the first week following photothrombotic stroke; B. Effects of lower-dose minocycline treatment (as defined in the “Treatment with minocycline” section) on forelimb function and cellular changes during the first week following photothrombotic stroke; C. Effects of higher-dose minocycline treatment (see “Treatment with minocycline” section) on forelimb function (up to 28 days) and cellular changes following photothrombotic stroke. The experimental protocols and rats used in each of the three investigations are summarized in Fig. 1.

For investigations of the effects of minocycline, rats were randomly pre-assigned to either minocycline treatment or vehicle treatment and all functional assessments, imaging, and data analyses were performed by investigators blinded to the treatment.

### Induction of focal ischemia by photothrombosis

An infarct was produced in the forelimb motor cortex using minor modifications of previously described procedures [29, 30]. Briefly, rats were anesthetized with 100 mg/kg ketamine and 10 mg/kg xylazine i.p. A cannula was inserted into the femoral vein and used to maintain anesthesia with additional injections of ketamine as required. The rat was placed in a stereotaxic frame, the scalp was retracted, and the surface of the skull cleared of periosteum and blood. A brass shim stencil with a rectangular window (3 × 5 mm) was positioned over the forelimb motor cortex at 1.5 mm anterior to bregma and 2.5 mm lateral to the midline. For initial characterization of the response to the infarct and studies of treatment with lower-dose minocycline (see below), the infarct was produced on the right side of the brain. For studies with the higher-dose of minocycline, the infarct was produced on the side contralateral to the limb used preferentially for a single pellet retrieval task (see below).

The skull was transilluminated for stroke induction using a 150 W light source (Intralux 5000; Volpi AG, Schlieren, Switzerland) equipped with a green bandpass filter (532.5–587.5 nm) and attached fiber-optic cable (6 mm diameter). Prior to each procedure, the light intensity was measured at the point of emission from the fiber-optic cable to ensure that it was at least 140,000 lx. In studies testing the effects of the higher dose of minocycline, a greater light intensity between 160,000 and 170,000 lx was used, which contributed to longer-lasting functional deficits. The fiber-optic cable was positioned directly over the window of the stencil and as close as possible to the skull without contacting it. Rose-bengal (10 mg/mL, 13 mg/kg) was injected via the femoral vein cannula at 200 μL/min and the light illuminated for 15 min.

### Treatment with minocycline

In the initial investigations into the effects of minocycline, the drug was administered i.p. at 50 mg/kg (10 mg/mL minocycline hydrochloride in phosphate-buffered saline (PBS), pH 7.4) or equivalent volume of PBS at 1 h and 24 h after stroke. The treatment regime was similar to other studies that have reported significant effects in suppressing microglial activation following stroke [20–22, 36]. Rats that were sacrificed at 24 h received only the first injection at 1 h after stroke. This treatment protocol will be referred to as “lower-dose minocycline treatment”.

In a subsequent series of experiments, a higher dosage treatment was used and is referred to as “higher-dose minocycline treatment”. The protocol was similar to treatments used in the studies of Chu et al. [25] and Kim et al. [37]. Minocycline (10 mg/mL in PBS) at 90 mg/kg or equivalent volume of PBS was administered i.p. at 1 h after stroke induction and then at 50 mg/kg at 12, 24, 36, and 48 h.

### Forelimb placing test

The forelimb placing response as described by Schallert and Woodlee [38] was used as a criterion for inclusion in the study and as the primary measure of recovery of forelimb motor response after stroke. Each rat was handled by an investigator for at least 10 min per day on three separate days leading up to the surgical induction of photothrombotic stroke. During these sessions, rats were placed on the testing platform for a few minutes and were given approximately five trials of each variant of the forelimb placing task.

For testing, the rat was held firmly by the torso in both hands such that all four limbs were hanging freely in space. The placing response in the forelimb was assessed by brushing the vibrissae of the rat against the test platform. The trial was scored as a success if the rat placed the forelimb on the platform. Three variants of the test were used. In the first, the rat was moved toward the platform so that both sets of vibrissae were stimulated. Subsequent tests involved stimulation of the vibrissae ipsilateral and then contralateral to the limb being assessed. Each forelimb was tested ten times with each of the three stimuli and a total success score out of 30 was calculated. Rats were initially tested at 3 h after stroke induction. Those rats with a score greater than 15 at this time were excluded from the study for the initial characterization of glial cell responses and investigations of the lower-dose minocycline treatment. For the higher-dose minocycline treatment, rats with a score greater than 5 were excluded, in an attempt to further restrict variability in the outcomes.

For studies of the effects of the higher dose of minocycline, neurological function was also evaluated using a cylinder test for forelimb asymmetry and a skilled reaching task, although only the results for the cylinder test have been presented (see below).

### Cylinder test for forelimb asymmetry

Forelimb use for weight support during rearing while rats were engaged in exploration in a Perspex cylinder (20 cm diameter, 30 cm height) was evaluated as previously described [29, 39]. The exploratory behaviour of rats was recorded with an Otek high-definition digital video camera. For measurements at 7 to 28 days, recording continued until the rat touched the cylinder walls approximately 100 times (median values for treatment groups at each time: 90 to 105; range for individual rats: 72 to 124 touches.) At 24 h, rats usually became less active within the first few minutes in the cylinder and fewer touches were recorded. (Median value of 37 for minocycline treated rats and 36.5 for vehicle-treated rats.) The percentage of affected limb usage was calculated as (the number of touches of the forepaw contralateral to the infarct + half the number of simultaneous placements/total paw placements) × 100.

### Single pellet skilled reaching test

Rats were trained to retrieve a pellet through a narrow slit in a single pellet reaching box and tested after stroke for total success and first attempt success in retrievals using the forelimb contralateral to the infarct as described previously [29, 40]. To facilitate training, rats were food restricted so that their body weight gradually reached 90 to 95% of that expected with free access to food. Rats were trained in daily 10-min sessions over 2 to 3 weeks up to the day prior to stroke induction. Initial training allowed the paw preference for this task to be identified. Subsequent training sessions led to improved success in the task. At the conclusion of the training period, median values for successful reaching for the two groups exceeded 50% on a 20-trial test. Rats were subsequently tested at 24 h and 7, 14, 21, and 28 days after stroke induction. However, the extent of recovery was highly variable between rats within groups. Thus, the results were not useful in detecting possible differences in functional recovery following treatment and have not been further discussed. Less variability on this test was seen previously when additional training in skilled reaching was provided during recovery [26]. The absence of such training was potentially a major contributor to the variability seen in the present study. Consistent with this possibility, our subsequent investigations of other treatments (manuscripts in preparation) indicated that the inclusion of further training after the stroke greatly improved consistency of responses within treatment groups enabling the identification of functional differences.

### Brain fixation and assessment of infarct volume

Rats were anesthetized with 100 mg/kg ketamine and 10 mg/kg xylazine i.p. and transcardially perfused at a rate of 60 mL/min with 10 g/L NaNO_2_ in 0.01 M sodium phosphate buffer (pH 7.4) followed after approximately 3 min by 200 mL 40 g/L paraformaldehyde in 0.1 M sodium phosphate buffer (pH 7.4). The brains were incubated in the same paraformaldehyde solution overnight at 4 °C*.* Brains were cryoprotected by phased transfer into 300 g/L sucrose in phosphate-buffered saline (PBS, pH 7.4) containing 0.2 g/L sodium azide. Coronal sections (20 μm) were prepared using a cryo-microtome (Leica Systems, NSW, Australia).

Slide-mounted sections at 0.5-mm intervals through the infarct were stained with 1 g/L cresyl violet in water acidified with acetic acid (60 μL/L) and imaged with a digital camera (Canon PowerShot A650 IS; Canon Australia, NSW, Australia), together with a calibration slide for scale. The area of the infarct in each section, as identified by pale staining, was measured using ImageJ version 1.49v [41]. Infarct volume between sections was approximated using the trapezoidal method (i.e., interval volume_a,b_ = 1/2 × (area_a_ + area_b_) × interval distance) and summed to give total infarct volume.

### Antibodies

Antibodies for immunohistochemistry to detect Iba1 (goat polyclonal; catalog number ab5976), vimentin (rabbit monoclonal; clone EPR3776; catalog number ab92547), and CD68 (rabbit polyclonal; catalog number ab125212) were from Abcam (Cambridge, UK), that for detecting glial fibrillary acidic protein (GFAP; rabbit polyclonal; catalog number G4546) was from Sigma-Aldrich (St Louis, Mo) and for NeuN (mouse monoclonal; clone A60; catalog number MAB377) was from Millipore-Chemicon (Merck, Darmstadt, Germany). Alexa-fluor-conjugated secondary antibodies were from Thermo Fisher Scientific (Waltham, MA, USA). For additional analysis of morphological changes in Iba1-immunolabeled cells, the antibody to detect Iba1 was from Wako Pure Chemical (Osaka, Japan; catalog #019-19741) and the biotinylated secondary antibody was from Jackson ImmunoResearch (West Grove, PA, USA; catalog #711-005-152).

For Western blot analysis, antibodies to detect GFAP (rabbit polyclonal; catalog number G3893) and actin (rabbit polyclonal; catalog number A2066) were from Sigma-Aldrich; those for vimentin (rabbit monoclonal; catalog number ab92547) were from Abcam and for neurocan (mouse monoclonal; clone 650.24; catalog number MAB5212) were from Millipore-Chemicon. A peroxidase donkey anti-rabbit IgG (catalog number: 711-035-712) from Jackson ImmunoResearch was used as secondary antibody.

### Immunohistochemistry

Free-floating brain sections were blocked and permeabilized in 0.3% Triton-X100, 5% donkey serum in PBS, pH 7.4 (blocking buffer) for 2 h at room temperature. The sections were then incubated overnight at 4 °C in primary antibodies diluted in blocking buffer (anti-Iba1 and anti-CD68, 1/1000, all other primary antibodies 1/500). The sections were washed four times in PBS before adding secondary antibodies (1/2000) and Hoechst 33258 (1/1000; Thermo Fisher Scientific) diluted in PBS and incubating for 2 h at room temperature on an orbital shaker. The sections were then washed four times in PBS before being mounted on glass slides in Prolong® Gold antifade mountant (Thermo Fisher Scientific) and protected with a coverslip.

### Image acquisition and analysis

**Fig. 2:**
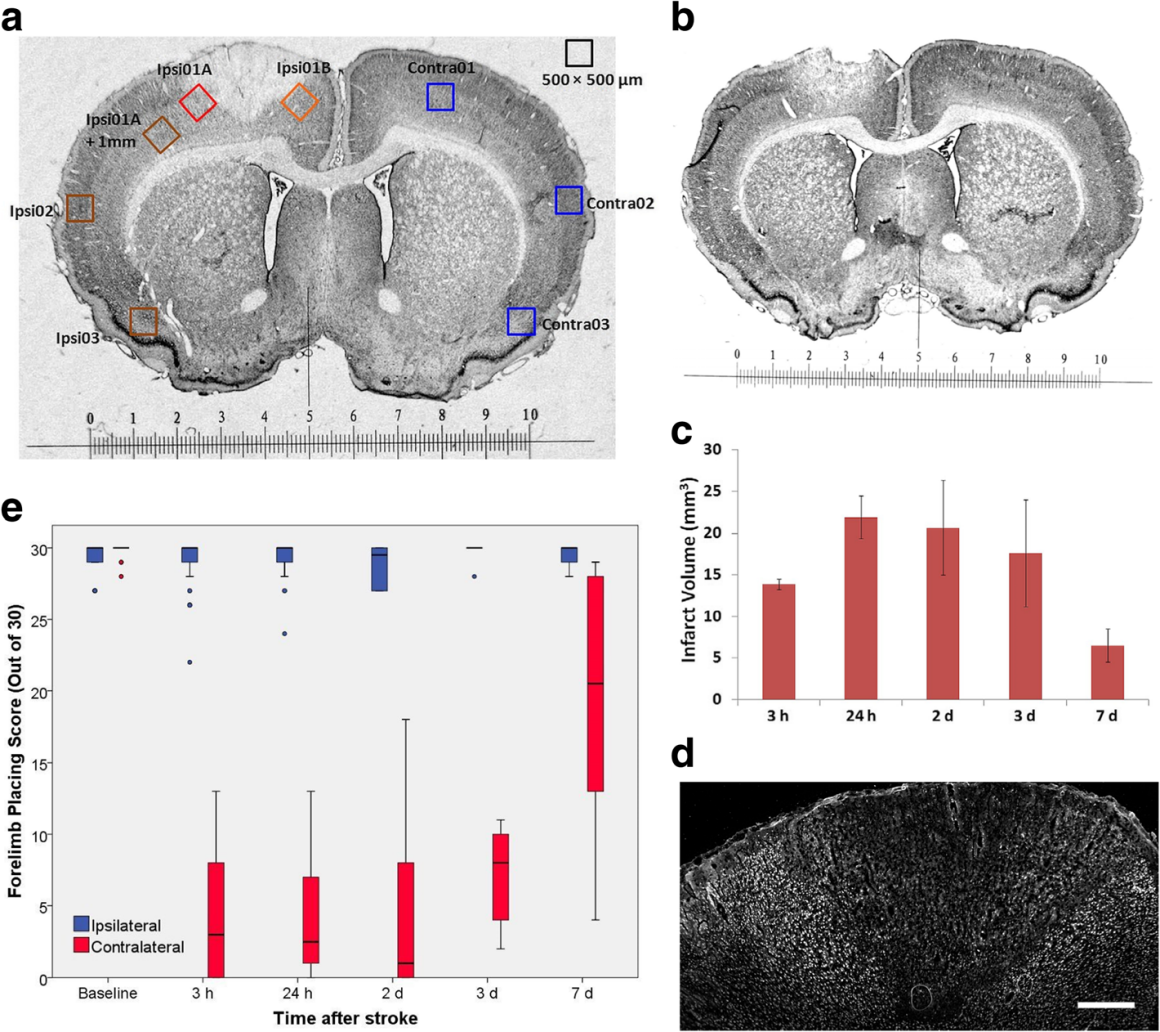
Tissue damage and functional changes following photothrombotic stroke. **a** Cresyl-violet stained coronal section of a rat brain at 24 h after induction of photothrombotic stroke showing the clearly defined infarct and the ROIs used for analysis of sections immunolabeled to detect Iba1. The scale is in millimeters. **b** Cresyl-violet stained coronal section at 3 h after stroke induction. **c** Infarct volume over 7 days after stroke induction. The infarct is fully developed by 24 h and then contracts substantially by 7 days (*n* = 4–6 per time point). **d** Coronal section of cerebral cortex immunolabeled for NeuN at 3 h after stroke induction (scale bar = 500 μm). **e** Box plot of forelimb placing scores following stroke. Outlier values are shown as circles; Blue = forelimb ipsilateral to infarct, Red = forelimb contralateral to infarct (*n* ≥ 5 per group)

For the initial characterization of changes in the microglial response, images were obtained from Iba1 and NeuN double-immunolabeled sections that incorporated the eight regions of interest (ROIs) as shown in Fig. 2a. This included ROIs on the lateral and medial edges of the infarct as well as 1 mm from the infarct and at more distant sites within the ipsilateral cerebral cortex. A single ROI from the contralateral hemisphere was used for comparison with the three sites close to the infarct. Two additional contralateral ROIs corresponded with the more distant sites in the ipsilateral cortex. The peri-infarct ROIs were placed immediately adjacent to the infarct within tissue where NeuN immunolabeling was preserved. For all investigations of immunolabeling, the reported results for each brain are the average of values from two sections separated by at least 0.5 mm.

For analysis of circularity and the area fraction of Iba1 immunolabeling, image stacks were scanned at 1 μm steps through the full thickness of the sections using a Leica TCS SP5 confocal microscope (Leica Microsystems Pty Ltd., NSW, Australia) with a × 20 objective lens. Image analysis was performed on maximum intensity projections generated in ImageJ version 1.49v. Background was reduced using the “subtract background noise” function with “rolling ball radius” set at 100 pixels and the sliding paraboloid function selected. Mean background fluorescence intensity was determined by averaging the fluorescence intensity at five sites that were devoid of cells. The threshold for subsequent image analysis was set at twice the mean background fluorescence intensity rounded down to the nearest integer.

ROIs with dimensions of 500 × 500 μm were analyzed. The ROIs were analyzed using the “Analyze Particles” function in ImageJ to obtain measures of “particle count,” “average size,” “% area,” and “circularity.” For these analyses, the particle size was set to a lower limit of 200 pixels. This size limit corresponds to circular particles of approximately 12 μm in diameter and was chosen to exclude cell debris or cells that were only partially within the section.

To complement the analysis of changes in morphology using ImageJ, additional sections from rats at 24 h and 7 days after stroke were labeled with Iba1 and a biotinylated secondary antibody [42]***.*** ROIs as shown in Fig. 2a were analyzed using Neurolucida morphology software (version 8; MBF Bioscience, VT, USA) to obtain measures of cell area, cell perimeter, and soma area of the Iba1-labeled cells [42]***.***

For an initial characterization of changes in the astroglial response, images were scanned from sections immunolabeled for both vimentin and NeuN double-immunolabeled sections using an Olympus IX71 fluorescence microscope with a 4-times objective lens. This analysis was limited to the peri-infarct tissue regions on either side of the infarct and a corresponding contralateral region. For the measurements, the peri-infarct ROIs extended 500 μm from the edge of the infarct, as identified from the NeuN labeling and covered the full depth of the cortical layer. Identical exposure settings were used to capture all images for vimentin immunofluorescence.

Sections double-immunolabeled with CD68 and NeuN were imaged using an Olympus AX70 fluorescence microscope with a 10-times objective lens. In preliminary analysis, CD68-positive cells in peri-infarct tissue were found to decrease sharply with distance from the infarct. Thus, counts of CD68-positive particles were obtained at two sites which are identified in the “Results” section as “peri-infarct” and “peri-infarct + 0.3 mm.” The peri-infarct tissue for this analysis extended 810 μm along the border of the tissue and 270 μm into the tissue with largely preserved NeuN labeling. This region was analyzed as the sum of three adjacent 270 × 270 μm boxes to facilitate alignment with the infarct border. The peri-infarct + 0.3 mm site was analyzed as a single rectangular box with one edge located 0.3 mm from the infarct adjacent to the peri-infarct tissue. It extended a further 270 μm into the intact tissue and was 810 μm in length. There was no overlap of tissue between the two regions that were examined.

### Western blots

Rats were decapitated under anesthesia induced by i.p. injection of ketamine (100 mg/kg) and xylazine (10 mg/kg) at 7 days after stroke induction. The brain was rapidly removed from the skull and the infarct was identified from the paleness of the tissue on the surface of the cortex. Cerebral cortex containing the infarct plus approximately 1.5 mm of surrounding tissue was dissected out for analysis.

The tissue samples were homogenized in a buffer containing 10 mM Tris, 1 mM EDTA, 0.32 M sucrose, pH 7.4, and a protease inhibitor cocktail (Sigma-Aldrich) using a Retsch TissueLyser (Qiagen, Chadstone, Vic, Australia) at a frequency of 30 Hz for 2 min. The homogenized samples were assayed for protein concentration using a Bio-Rad DC protein assay kit (Bio-Rad Laboratories Gladesville, NSW Australia). Before gel electrophoresis, samples used for neurocan blots were pre-treated by incubating with chondroitinase ABC (1 unit/mL) in 0.2 M Tris (pH 8.0) containing 0.3 M sodium acetate for 3 h at 37 °C [43].

Samples (20 μg protein in 20 μL) and molecular weight standards (Precision Plus Protein™ Dual Color Standards, #1610394; Bio-Rad Laboratories) were loaded for gel electrophoresis (4–20% Criterion™ TGX Stain-Free™ Protein Gel, #5678094; Bio-Rad Laboratories) then transferred to PVDF membrane by electrophoresis (Trans-Blot® Turbo™ Transfer System with RTA Midi LF PVDF Transfer Kit; Bio-Rad Laboratories). The PVDF membranes were pre-treated by incubating in 5% skim milk and 0.1% Tween 20 in PBS (pH 7.4) for 1 h at room temperature. All antibodies were diluted (1:1000 for primary antibodies and 1:2000 for secondary antibodies) in 2.5% skim milk and 0.05% Tween 20 in PBS (pH 7.4). The membranes were incubated in primary antibodies for 30 min at room temperature with regular manual agitation and then overnight at 4 °C on an orbital shaker. They were then incubated in secondary antibody for 2 h at room temperature. The bands were developed by incubation in 5 mL ECL substrate (Clarity™ Western ECL Blotting Substrate; Bio-Rad Laboratories) for 5 min at room temperature.

Chemiluminescence from the blots was captured digitally on a Gel Doc™ EZ Imager (Bio-Rad Laboratories) and the band intensities were analyzed using the Carestream Molecular Imaging Software (Version 5.0; Carestream Health, NY, USA). The intensity of vimentin or GFAP bands were expressed relative to the bands for actin. The intensity of the band for full-length neurocan was expressed as a ratio to a fragment of this protein with a molecular weight of approximately 150 kDa, that was readily detectable in all samples. In mature brain, neurocan is almost completely present as fragments generated by proteolysis [44]. In response to injury, the content of full-length neurocan increases greatly in neighboring brain tissue, whereas the expression of the truncated form is usually little changed [45–47].

### Statistical analysis

Data from tests of neurological function are presented as box plots. All other data are shown as mean ± standard deviation. Statistical analyses were performed using the SPSS software package (IBM SPSS Statistics for Windows. Version 23.0; IBM Corp., Armonk, NY, USA). For assessing possible differences in recovery of function using the forelimb placing test, the area under the plot of test performance versus time was calculated including all points between the assessment at 3 h after stroke induction and the final assessment prior to euthanasia. For the cylinder test, the area under the curve between 24 h and 28 days was measured. Values were compared using a non-parametric Mann-Whitney test. For other data, which involved comparisons between multiple groups, analysis of variance was used followed by post-hoc testing with Tukey’s HSD test when statistically significant differences were identified. Student’s *t* test was used for comparisons between two groups. For all analyses, differences with *p* < 0.05 were considered statistically significant.

## Results

### Characterization of peri-infarct microglial responses following stroke

Formation of the infarct was already well advanced at 3 h after induction of the photothrombotic stroke based on the volume of tissue exhibiting pale staining with cresyl violet (Fig. 2b). The infarct volume reached a maximum by 24 h and had contracted greatly by 7 days (Fig. 2c). Immunolabeling for the neuronal marker, NeuN, was largely lost in the infarct by 3 h after stroke induction (Fig. 2d). Thus, tissue on the edge of the infarct retaining high-density NeuN labeling could be readily delineated across the full range of times investigated.

Marked deficits in the function of the forelimb contralateral to the infarct had developed within 3 h of stroke induction as assessed from the forelimb placing test and remained greatly impaired over the first 3 days (Fig. 2e). By 7 days, recovery was substantial but incomplete in most rats. The forelimb ipsilateral to the infarct produced near maximal scores at baseline and at all times tested after stroke (with a median score of 30 at all time points except for 2 days when the median score was 29).

**Fig. 3:**
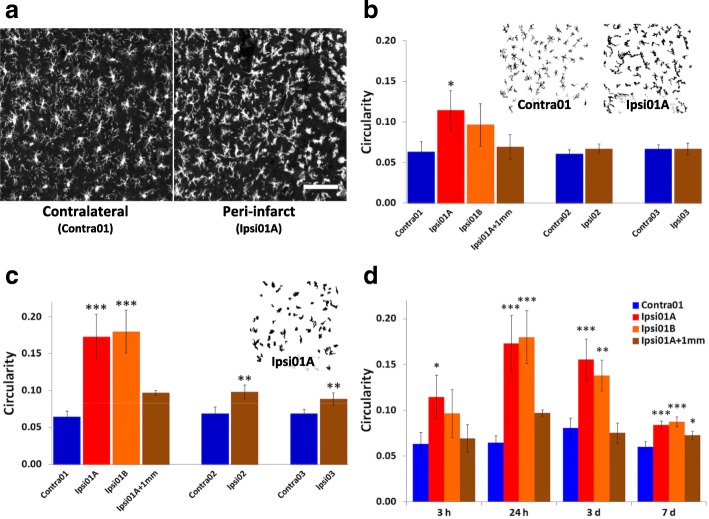
Circularity of Iba1-positive cells after photothrombotic stroke. This parameter was measured in ROIs as defined in Fig. 2a. **a** Comparison of Iba1-immunolabeled cells in peri-infarct tissue (Ipsi01A) and equivalent tissue (Contra01) in the hemisphere contralateral to the infarct at 3 h after stroke induction. The scale bar = 100 μm. **b** Regional differences at 3 h. Insets show examples of processed images of Iba1-positive cells at an ROI adjacent to the infarct (Ipsi01A) and in corresponding contralateral tissue (Contra01). **c** Regional differences at 24 h. Inset shows an example of a processed image at Ipsi01A. **d** Time course of changes in peri-infarct regions (Ipsi01A and Ipsi01B), tissue 1 mm from the infarct (ipsi01A + 1 mm) and corresponding contralateral tissue (Contra01) over the first 7 days. For panels **b**–**d**, *n* = 4 for each data point; *p < 0.05, **p < 0.01, ***p < 0.001 compared with the corresponding contralateral region (one-way ANOVA with Tukey’s HSD post-hoc test)

Alterations of microglial morphology with loss of ramification of cell processes were already apparent in peri-infarct tissue at 3 h after stroke induction (Fig. 3a). These changes contributed to a significant increase by more than 80% in microglial circularity in one of the peri-infarct ROIs, Ipsi01A when compared with equivalent contralateral tissue (Fig. 3b). Circularity was not significantly altered at other ROIs in the ipsilateral cortex at this time. In both peri-infarct regions at 24 h, circularity had increased more than 2.5-fold above values in the contralateral tissue (Fig. 3c). Increases in circularity of peri-infarct microglia compared with the contralateral regions largely persisted at 3 days and remained elevated, albeit less so, at 7 days (Fig. 3d). Smaller increases were also seen at 24 h in the damaged hemisphere at sites up to several millimeters from the infarct (Ipsi02 and Ipsi03; Fig. 3c). A significant increase (*p* < 0.01) was still present in Ipsi02 (0.079 ± 0.002) compared with corresponding contralateral tissue (0.065 ± 0.003) at 3 days but no significant difference was seen in Ipsi03 at this time. Neither of these ROIs differed significantly from equivalent contralateral tissue at 7 days. However, a small increase was observed in tissue 1 mm from the infarct (Fig. 3d).

**Fig. 4:**
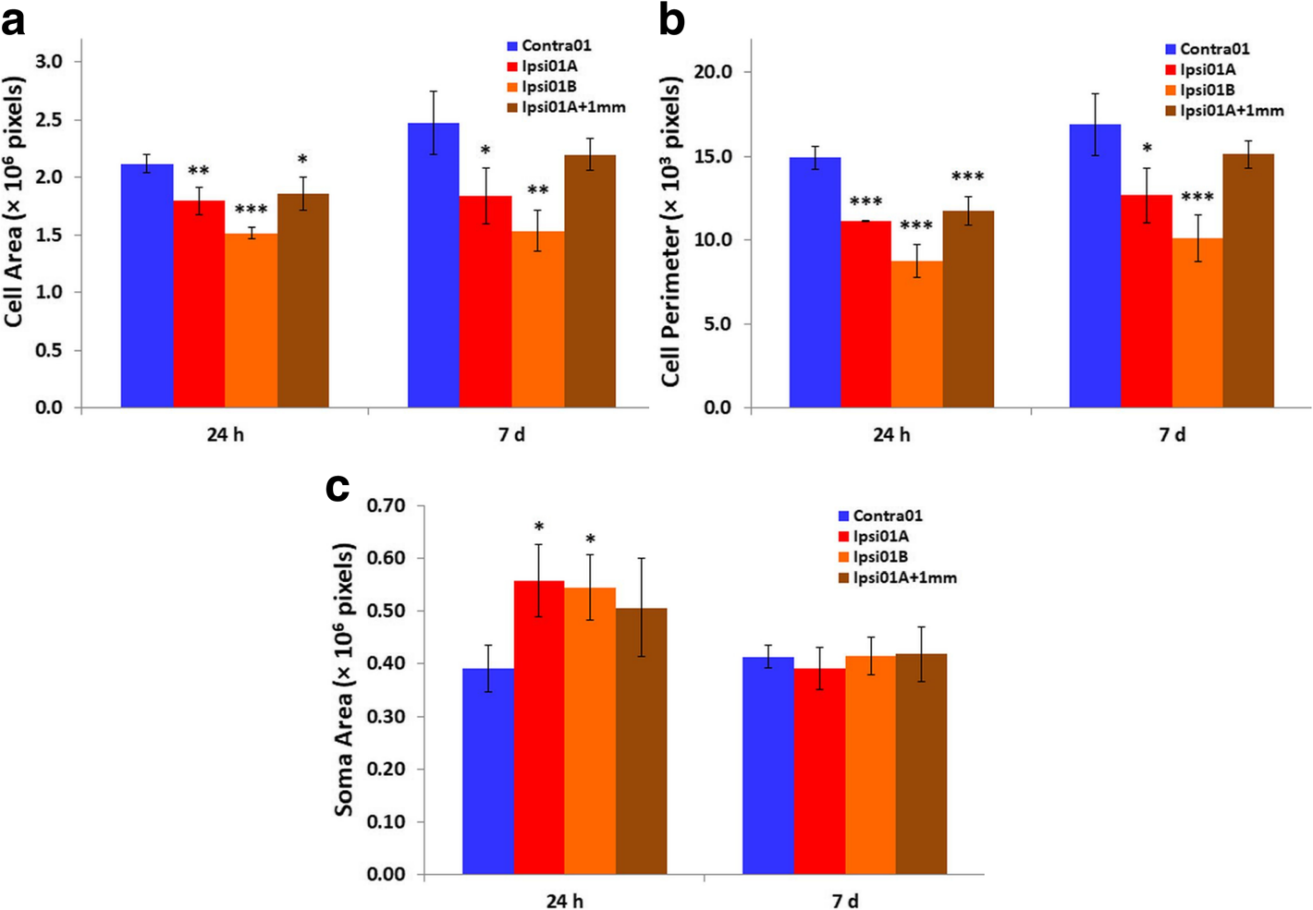
Additional morphological features of Iba1-positive cells at 24 h and 7 days after photothrombotic stroke. **a** Cell area, **b** cell perimeter, and **c** cell soma area. For panels **a**–**c**, *n* = 3 (7 days) or 4 (24 h); *p < 0.05, **p < 0.01, ***p < 0.001 compared with the corresponding contralateral region, contra01 (one-way ANOVA with Tukey’s HSD post-hoc test). The ROIs that were analyzed are identified in Fig. 2a

To further characterize the morphological responses of the microglia and aid in interpreting the findings based on cell circularity, three additional features of the Iba1-immunolabeled cells (cell area, cell perimeter, and area of the cell soma) were measured in separate sections from the same rats at 24 h and 7 days (Fig. 4a–c). Cell area and perimeter were significantly decreased in the two peri-infarct regions at both 24 h and 7 days (Fig. 4a, b). These measures were also significantly decreased at two of the sites more distant from the infarct (ipsi01A + 1 mm, ipsi03) at 24 h but not 7 days (Fig. 4 a, b; Additional file 1: Figure S1 A and B) largely consistent with the pattern of responses detected with circularity. The third more distant region (ipsi02) was not significantly different on these measures from the equivalent contralateral region at 24 h but showed increases at 7 days (Additional file 1: Figure S1 A and B). The area of the soma was significantly increased at 24 h in the peri-infarct tissue (Fig. 4c). This measure was not significantly different at 7 days in these tissue regions or at any time point in the more distant regions (Fig. 4c; Additional file 1: Figure S1).

**Fig. 5:**
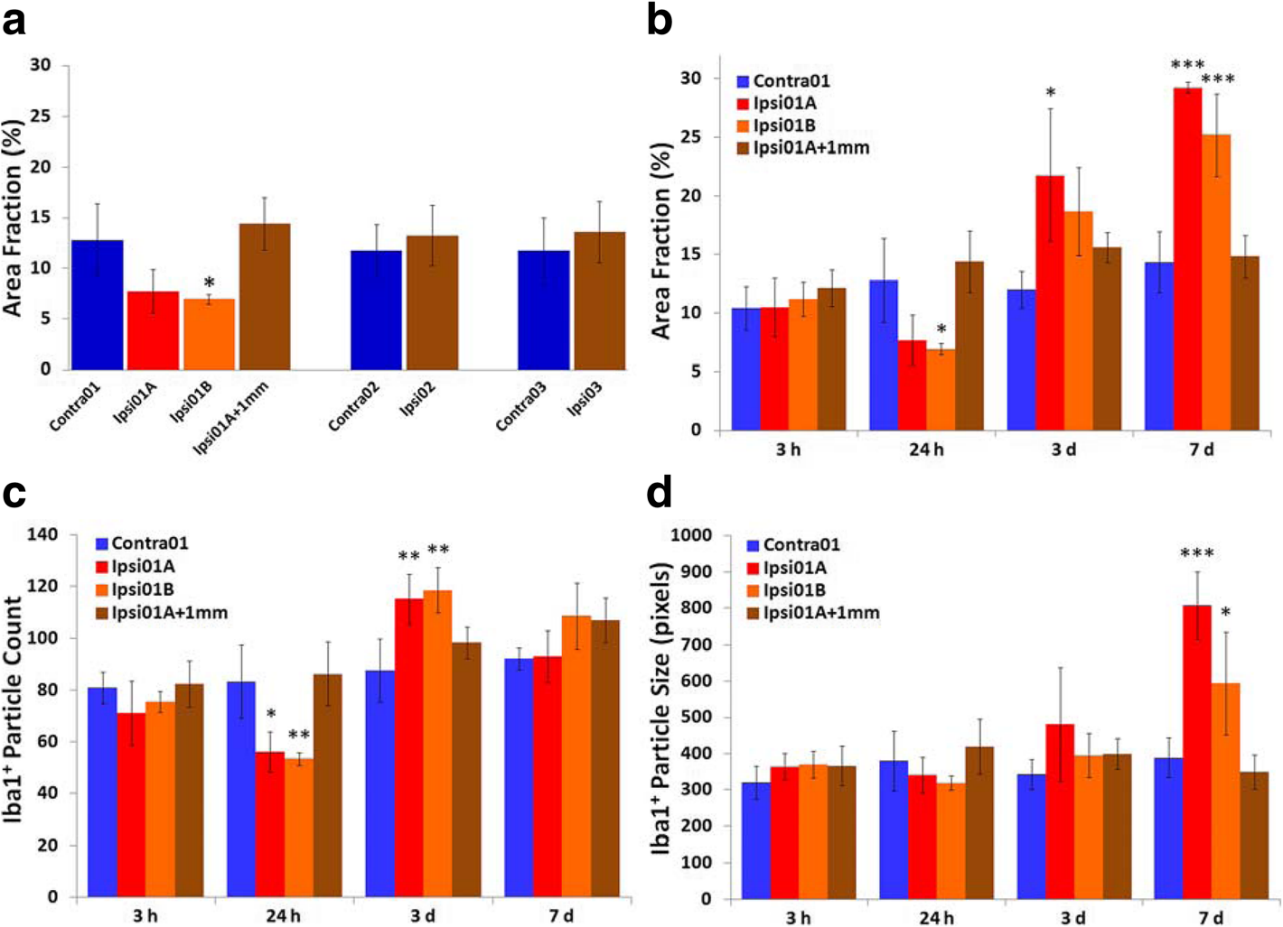
Changes in the pattern of Iba1-immunolabeling after photothrombotic stroke. **a** Regional differences in area fraction at 24 h. **b–d** Changes in **b** area fraction, **c** Iba1-positive particle count, and **d** Iba1-positive particle size in peri-infarct tissue, tissue 1 mm from the infarct and corresponding contralateral tissue between 3 h and 7 days. For all panels, *n* = 4 per data point; *p < 0.05, **p < 0.01, ***p < 0.001 compared with Contra01 (one-way ANOVA with Tukey’s HSD post-hoc test). At 24 h, there was also a significant difference between each of the peri-infarct regions and tissue located 1 mm from the infarct (at ipsi01a + 1 mm) for area fraction (Ipsi01a: *p* < 0.05; Ipsi01b: *p* < 0.01) and particle count (*p* < 0.01 for each of the peri-infarct regions). The ROIs that were analyzed are identified in Fig. 2a

The area fraction of Iba1 immunolabeling was not significantly changed in any region of the lesioned hemisphere at 3 h after stroke onset. At 24 h, area fraction in the peri-infarct ROI, ipsi01b, was significantly reduced compared with corresponding tissue in the contralateral region (Fig. 5a). The other peri-infarct ROI, ispi01a, showed a trend toward a similar decrease but the difference was not statistically significant (*p* = 0.056). Both of the peri-infarct ROIs had a significantly lower area fraction (*p* < 0.05) compared with tissue located 1 mm from the infarct (Fig. 5a). Additional analysis of the number of Iba1-labeled particles in the peri-infarct tissue showed a significant decrease (by 34%) in the two peri-infarct regions compared with the equivalent contralateral tissue (Fig. 5c). Some swelling of the affected tissue was observed (Fig. 2a). Although this could contribute to the reduction in area fraction, it was very much less than the 50% increase in volume in peri-infarct tissue that would fully account for the observed decrease in Iba1 immunolabeling. A loss of cells due either to cell death or a migration of cells out of the peri-infarct tissue is more likely to account for most of the change in area fraction of Iba1 labeling at 24 h.

By 3 days, Iba1 labeling in peri-infarct tissue was markedly increased compared with the contralateral hemisphere and increased further by 7 days (Fig. 5b). Particle counts at 3 days were also increased in peri-infarct tissue indicating that changes in total cell number were a major component of the increase in area fraction in the peri-infarct tissue (Fig. 5c), In contrast, the particle count at 7 days was not significantly different from the equivalent contralateral tissue (Fig. 5c) despite the continuing increase in area fraction. This finding points to a limitation in the use of particle counts at 7 days. At this time, but not the earlier time points, the measure of particle size was increased in the peri-infarct tissue (Fig. 5d). This change did not result from increases in the size of individual cells. Rather it reflected overlap between neighboring cells as the numbers markedly increased such that many cells were not separately identified in the analysis. In contrast to the large changes in the peri-infarct regions, tissue 1 mm from the infarct and at more distant ipsilateral sites (Ipsi02 and Ipsi03) showed no significant differences in area fraction compared with corresponding regions in the contralateral hemisphere at any of the time points investigated (Fig. 5a, b).

The findings from the initial characterization indicated that the circularity and area fraction of Iba1 immunoreactivity provide useful insights into important aspects of the responses of microglia (and other Iba1-positive cells) in the peri-infarct tissue following photothrombotic stroke. For subsequent studies examining the effects of minocycline on recovery from photothrombotic stroke, changes in these properties were investigated only in a single peri-infarct region (equivalent to Ipsi01A in Fig. 2a) as the responses in the two peri-infarct regions were similar and changes outside the peri-infarct region were either not seen or were smaller and more short-lived.

### Lower-dose minocycline treatment

**Fig. 6:**
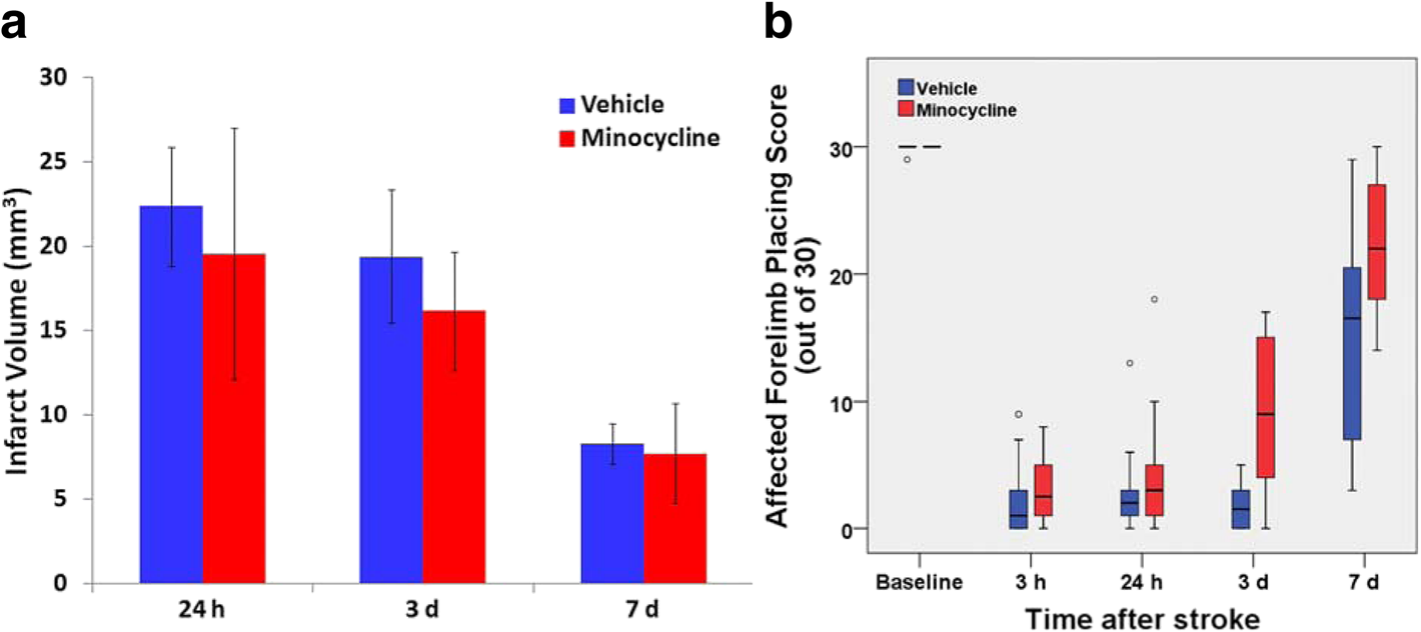
Effects of lower dose minocycline treatment on infarct volume and forelimb function after photothrombotic stroke. **a** Infarct volume: Two-way analysis of variance revealed no significant effect of the minocycline treatment but a significant effect of time after stroke (*p* < 0.01; *n* = 4–8 per group). **b** Box plot of results from the forelimb placing test. Outliers are indicated with open circles. *n* = 6 per group for all times except 3 h which shows the pooled data for rats in each of the subsequent groups. Rate of recovery was faster at 3 days *p < 0.05 (Minocycline vs vehicle, Mann-Whitney *U* test analysis of area under curve for those rats tested at both 3 h and 3 days). No significant differences were detected at other times

Treatment with minocycline had no effect on infarct volume measured at 24 h to 7 days after stroke (Fig. 6a). However, the treatment produced a more rapid recovery of function as seen from a significantly better forelimb placing response at 3 days (Fig. 6b). By 7 days, recovery was substantial in both groups and the difference between the groups was not statistically significant. Placing scores for the limb ipsilateral to the infarct were preserved with median scores of 30 for both treatment groups at all times except at 3 h when median scores were 28.5 and 29 for the two groups.

**Fig. 7:**
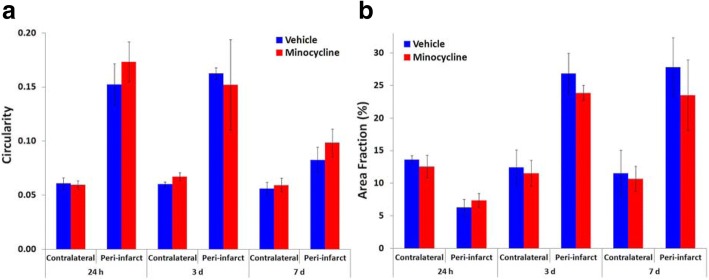
Effects of lower dose minocycline treatment on responses of Iba1-positive cells after photothrombotic stroke. **a** Circularity and **b** area fraction of Iba1-positive cells at 24 h, 3 days and 7 days following photothrombotic stroke (*n* = 4 to 8 per group). Two-way analysis of variance detected highly significant differences between the contralateral and peri-infarct regions for both circularity and area fraction at all three time points (*p* < 0.001). However, there were no significant effects of treatment on these parameters

The changes in microglial circularity and area fraction of Iba1 immunolabeling in the vehicle-treated group were consistent with those seen in the initial characterization. Circularity was maximally increased at 24 h, remained similarly elevated at 3 days, and was then partially decreased by 7 days (Fig. 7a). For area fraction, the initial decrease in peri-infarct tissue at 24 h to less than 50% of the contralateral regions (Fig. 7b) was even more pronounced than in the initial characterization. In contrast to predictions based on previous reports of the action of minocycline (see Introduction), this treatment had no significant effect on either microglial circularity or area fraction of immunolabeling between 24 h and 7 days after stroke (Fig. 7a, b).

### Higher-dose minocycline treatment

The ability of minocycline to influence the activation of peri-infarct microglia and recovery after stroke was further assessed in rats that received a more intensive treatment with this drug and were monitored for recovery of neurological function for 28 days after stroke. These rats were injected with an initial higher minocycline dose (90 mg/kg) and received subsequent injections of 50 mg/kg at 12, 24, 36, and 48 h. For this part of the study, a moderately higher intensity of the light source was used to induce thrombosis. The inclusion criteria were also made more stringent such that rats with a placing score above 5 at 3 h were excluded from further study.

The higher-dose minocycline treatment again did not significantly affect infarct volume as assessed at 3 days after stroke induction (vehicle-treated: 17.20 ± 4.88; minocycline-treated: 18.56 ± 5.26 mm^3^, mean ± SD, *n* = 6–7).

**Fig. 8:**
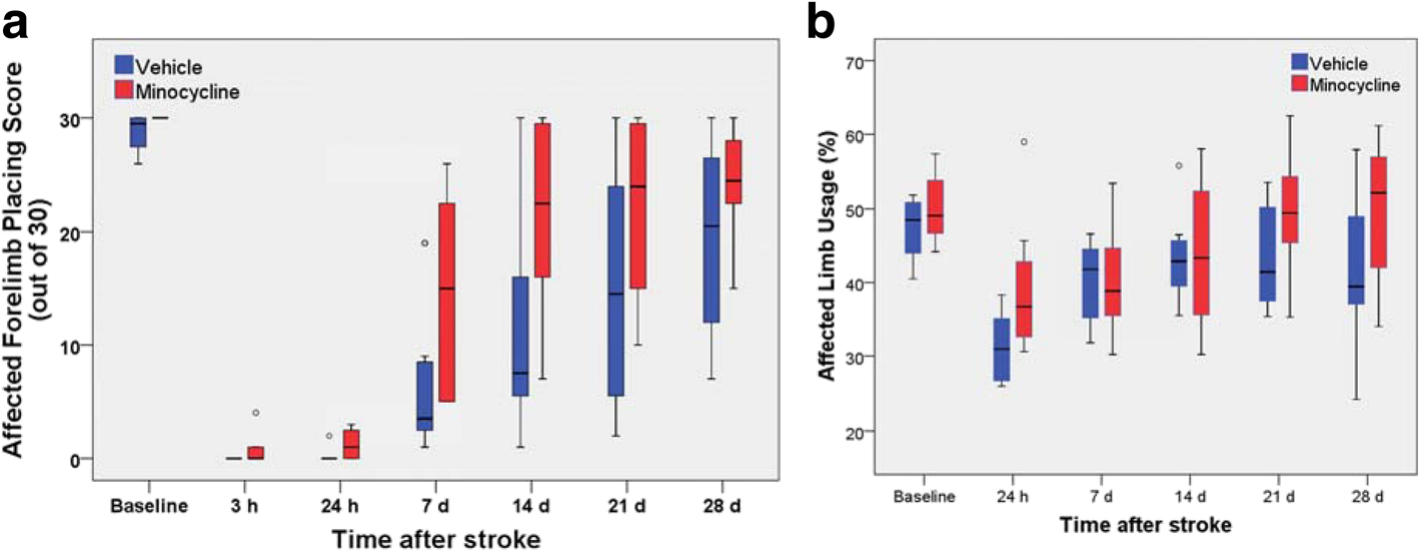
Effect of higher-dose minocycline treatment on recovery of neurological function after photothrombotic stroke. Box plots of dysfunction and recovery up to 28 days after photothrombotic stroke in **a** the forelimb placing test and **b** the cylinder test. Outliers are shown as open circles (*n* = 8 per group). Recovery in the forelimb placing test was more rapid in minocycline-treated rats (*p* < 0.05; Mann-Whitney *U* test comparison of area under curve for time points between 3 h and 28 days.) There was no significant difference in recovery between 24 h and 28 days for performance in the cylinder test

The modified procedures for stroke induction and/or more stringent exclusion criteria led to longer-lasting deficits in the forelimb placing test in the vehicle-treated group with most animals still exhibiting impairment at 28 days (Fig. 8a). Rats treated with minocycline recovered more rapidly on this test (*p* < 0.05), with differences particularly prominent at 7 and 14 days. There were no significant changes in placing scores following stroke induction for the forelimb ipsilateral to the infarct (with median group scores of 28 to 30 for all time points for both minocycline-treated and vehicle-treated rats).

Stroke also induced deficits in spontaneous use of the forelimb contralateral to the infarct as assessed from the cylinder test at 24 h after induction but these were less severe than the initial changes in forelimb placing (Fig. 8b). The rate of recovery in the cylinder test task did not differ significantly between the two treatment groups.

**Fig. 9:**
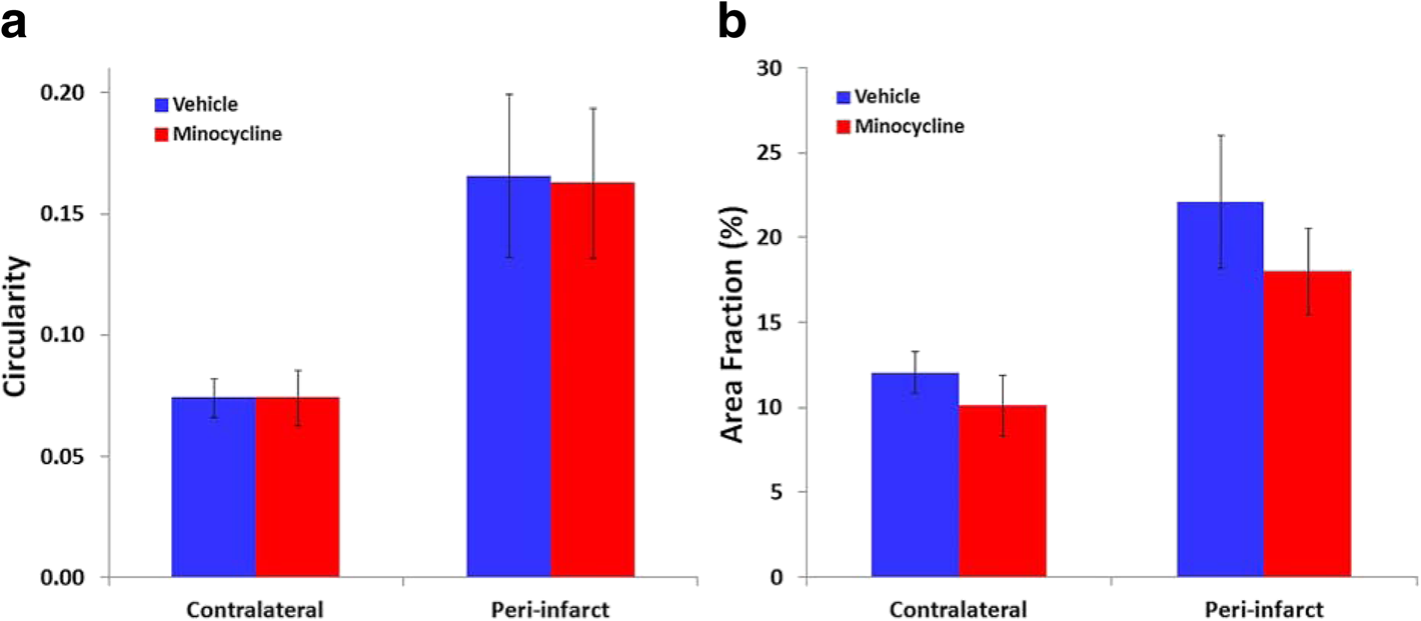
Effect of higher-dose minocycline treatment on Iba1-positive cells at 3 days after photothrombotic stroke. **a** Circularity and **b** area fraction of immunolabeling (*n* = 6 to 7 per group). Two-way analysis of variance revealed a statistically significant difference between the hemispheres for both circularity and area fraction (*p* < 0.01) and a significant effect of the minocycline treatment on Iba1 area fraction (*p* < 0.05) but not on circularity. For area fraction, there was no significant interaction between hemisphere and minocycline treatment

The more intense treatment with minocycline had no significant effect on circularity of Iba1-positive cells when assessed at 3 days (Fig. 9a). However, small reductions were seen in area fraction in the minocycline-treated group at this time (Fig. 9b). Similar decreases were found in both hemispheres indicating that this was a generalized change induced by the minocycline and did not primarily reflect changes in the specific microglial response to infarct formation.

**Fig. 10:**
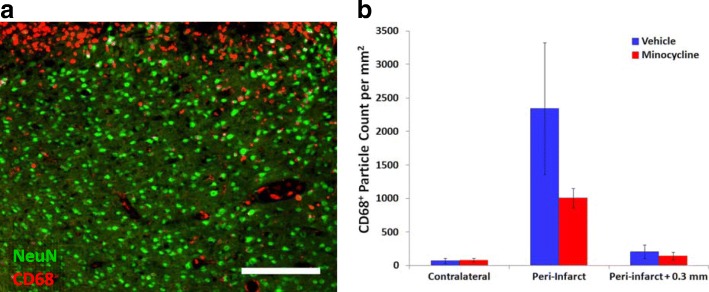
Effect of higher-dose minocycline treatment on CD68-immunolabeled cells at 3 days after photothrombotic stroke. **a** Representative image of CD68 (red) and NeuN (green) double-immunolabeled coronal section at the infarct boundary of a vehicle-treated rat. The infarct is visible in the upper part of the image. The scale bar = 200 μm. **b** Effects of the higher-dose minocycline treatment on CD68-positive cells within the peri-infarct tissue at 3 days following photothrombotic stroke (*n* = 6 to 7 per group). The CD68-positive particle count was assessed within 0.3 mm of the infarct (Peri-infarct) and between 0.3 and 0.6 mm from the infarct (Peri-infarct + 0.3 mm). Two-way analysis of variance revealed a highly significant effect of both treatment (*p* < 0.01) and tissue location (*p* < 0.001) on CD68-positive particle count at 3 days after stroke. There was also a strong interaction between the two factors (*p* < 0.001) reflecting the larger decrease in particle density induced by minocycline in the tissue immediately adjacent to the infarct compared with the other regions examined

The number of cells immunolabeled for CD68, a marker of phagocytic activity in microglia and macrophages, were also measured at 3 days as CD68-positive cells have previously been reported to be altered by minocycline treatment (see “Discussion” section). At this time, cells expressing CD68 were numerous within the infarct (Fig. 10a). The density of these cells in peri-infarct tissue was much lower than in the infarct and was largely restricted to a region immediately adjacent to the infarct. Compared with the pattern of change in morphology and density of Iba1-positive cells, the distribution of the CD68-positive cells appeared to be much more tightly associated with the rim of the infarct.

Minocycline treatment markedly decreased the numbers of CD68-positive cells by more than 50% in peri-infarct tissue that was within 0.3 mm of the infarct (Fig. 10b). At distances of more than 0.3 mm, there were many fewer CD68-positive cells with similar numbers in the two treatment groups.

**Fig. 11:**
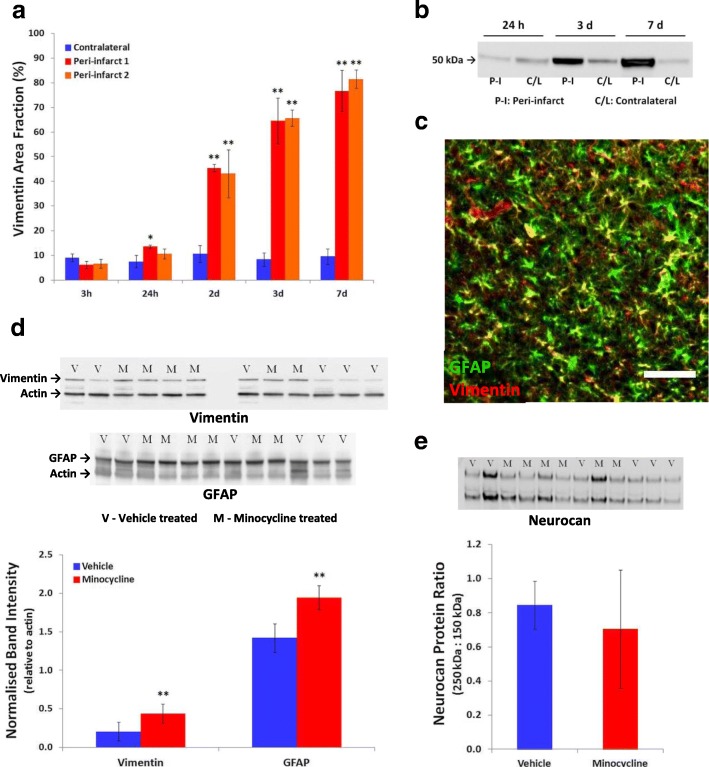
Effects of higher-dose minocycline treatment on markers of reactive astrogliosis after photothrombotic stroke. **a**, **b** A large increase in vimentin content in peri-infarct tissue over the first 7 days following photothrombotic stroke in untreated rats was detected by **a** area fraction of vimentin-positive cells and **b** Western blotting. The area fraction of vimentin immunolabeling was markedly increased by 2 days and further increased at 3 and 7 days after stroke (*n* = 4 per group at each time); *p < 0.05, **p < 0.01 vs. corresponding contralateral region (one-way ANOVA with Tukey’s HSD). **c** Double immunolabeling of GFAP and vimentin in peri-infarct tissue at 3 days after photothrombotic stroke. Vimentin (red) is expressed almost exclusively in cells also expressing GFAP (green). Yellow shows colocalization. The scale bar = 100 μm. **d**–**e** Effect of higher-dose minocycline treatment on expression of astroglial proteins in samples containing infarct plus peri-infarct tissue at 7 days following photothrombotic stroke (*n* = 6 per group). **d** The intermediate filament proteins, vimentin and GFAP. **e** Neurocan. The band intensities for vimentin (54 kDa) and GFAP (50 kDa) were normalized to actin (45 kDa). There was a significant effect of minocycline treatment on both vimentin and GFAP content (**p < 0.01). The intensity of bands for full-length neurocan (approx. 250 kDa) were determined relative to that of a proteolytic fragment of this protein (approx. 150 kDa). No statistically significant effects were detected in the neurocan protein ratio in tissue from the minocycline-treated rats compared with the vehicle-treated rats

Minocycline could also potentially modify downstream responses in peri-infarct reactive astrogliosis that are at least partly initiated by microglia [16]. This possibility was initially assessed from measures of the content of the cytoskeletal proteins, GFAP and vimentin, using Western blotting of tissue samples containing the infarct and peri-infarct tissue. Immunohistochemical detection of these proteins in our samples revealed only low levels of residual expression in the infarct. Thus, the major portion of their content in the sampled tissue was derived from the marked increase in expression in peri-infarct cells. Vimentin was included in this analysis because of the potential to provide a more sensitive indicator of responses of reactive astrocytes than GFAP. In the parenchyma of the normal mature brain, vimentin is expressed by endothelia and perhaps other cells surrounding blood vessels but is very rarely expressed by astrocytes [48, 49]. Initial analysis of vimentin expression based on both area fraction of vimentin immunolabeling in tissue sections (Fig. 11a) and Western blots (Fig. 11b) revealed large increases in peri-infarct tissue at 3 days after stroke and further increases at 7 days to values that were many times greater than in equivalent contralateral tissue. The vimentin immunolabeling in the peri-infarct tissue co-localized almost completely with GFAP labeling (Fig. 11c).

The content of both vimentin and GFAP at 7 days after stroke induction was substantially increased (to 210% and 137% respectively) in the samples from minocycline-treated rats (Fig. 11d) compared with those injected with vehicle, indicating an increase in at least some aspects of reactive astrogliosis. The content of full-length neurocan, a proteoglycan that is largely produced by astrocytes and increases in response to brain tissue damage [45–47], was also investigated. In normal mature brain, this protein is almost all in the form of two fragments derived by proteolysis from the full protein. The content of the full-length protein increases markedly in peri-infarct tissue following stroke [46]. In our study, the ratio of full-length neurocan to a 150-kDa fragment of this protein was not significantly different between the minocycline-treated and vehicle-treated groups (Fig. 11e).

## Discussion

The study provides evidence that early post-stroke minocycline treatment can improve recovery of aspects of neurological function in rats and that this was associated with increases in indicators of reactive astrogliosis and decreases in cells expressing CD68, a marker of microglia/macrophage phagocytosis. However, the minocycline treatment produced little, if any, suppression of peri-infarct changes in microglial morphology or the density of immunolabeling of these cells even though modifications to these properties were predicted based on previous investigations of the actions of this drug.

In addition, the characterization of microglial responses to cortical stroke identified a previously unreported decrease in the area fraction of Iba1 immunolabeling in peri-infarct tissue at 24 h that was primarily due to fewer cells in this tissue. The decrease was most likely the result of early migration of microglia into the infarct and/or localized death of these cells. It was followed by marked increases in Iba1-positive cells in peri-infarct tissue at 3 to 7 days. Morphological changes in these cells in peri-infarct tissue were readily apparent within 3 h of stroke and could be detected from increases in cell circularity. The increases in circularity became more prominent by 24 h and were transiently seen at sites more distant from the infarct.

Minocycline-treated rats showed improved recovery in a forelimb placing test, which was the primary measure of forepaw function in this study. This measure requires no specific training of the rats and involves a motor response to sensory stimulation. The improvement occurred in the absence of changes in infarct volume and is broadly consistent with reports of improved outcome without changes in infarct volume following minocycline treatment in several other studies [25–27]. However, treatment was limited to the first 2 days after stroke in our study contrasting with these earlier studies showing improved recovery [25–27], as well as some investigations of the neuroprotective effects of minocycline in rodents [20, 22] and in humans [50]. Thus, the findings suggest that cellular changes critical for recovery are initiated in the early stages after infarct formation and that they can be modulated by minocycline treatment.

Long-term minocycline treatment has been shown previously to moderately improve use of the affected forepaw during spontaneous movement as assessed using a cylinder test [27]. In contrast, no significant effect on performance in this test was detected in our study. The differential effects on performance in the forelimb placing test and cylinder test could be the result of a greater sensitivity of the placing test because the initial stroke-induced deficits were larger. A similar finding of significant improvement in a paw placing test but not in a second test of neurological function (involving skilled reaching) was seen with environmental enrichment following stroke in rats [51]. Thus, it is also possible that plasticity can be more readily modified by specific interventions in some components of the sensorimotor neural pathways that are involved in the forelimb placing test.

Iba1 immunolabeling was selected as the primary means to assess changes in microglia and macrophages in the peri-infarct tissue because it allowed time-dependent features of activation of these cells to be assessed at sites adjacent to the infarct. The peri-infarct tissue of interest was reproducibly identified based on the pattern of co-labeling with the neuronal marker, NeuN. The activation of microglia and macrophages in peri-infarct tissue has also commonly been assessed in previous studies based on the detection of changes in cell-selective immunolabeling (see for example: [8, 9, 34, 35, 52]). However, most of the previous studies have only provided qualitative descriptions of the pattern of changes in markers of microglia and macrophages. The detailed analysis of morphology by Morrison and Filosa [8] and assessments of the numbers of proliferating Iba1-positive cells by Li et al. [35] are notable exceptions, although the former study reported on changes in peri-infarct tissue only at 8 and 24 h after temporary focal ischemia.

The treatment with lower-dose minocycline delivered in the first 24 h after stroke did not produce significant differences in either circularity or Iba1 area fraction during the first 7 days. The higher-dose treatment, which was administered for the first 48 h after stroke, did produce changes in Iba1 area fraction. However, this was a small effect and similar changes were seen in tissue from the contralateral hemisphere. Thus, our findings suggest that minocycline did not produce gross alterations in these key responses to stroke in the Iba1-positive cells in peri-infarct tissue. In other studies of the effects of minocycline on responses to tissue damage in the brain, including early influential investigations in models of focal and global ischemia [20, 53], minocycline was reported to greatly limit changes in microglial morphology or cell distribution (as assessed qualitatively). The different response to focal ischemia compared with that in the present study is probably explained, at least in part, because minocycline produced decreases in cell loss and infarct size in many of the earlier investigations of stroke and such protective effects can secondarily reduce microglial responses to ischemic damage [54, 55]. Other factors could have contributed to the different outcome including the initiation of treatment before stroke induction in some previous studies and the lack of procedures to ensure consistent sampling of peri-infarct tissue as was achieved with the co-labeling for NeuN in our investigations.

In contrast to the limited effects of minocycline on the measures of Iba1-immunolabeling in peri-infarct tissue, there was a marked reduction at 3 days after stroke induction in cells expressing CD68, a protein usually associated with microglia and macrophages that are involved in phagocytosis. This raises the possibility that CD68-positive cells might have effects that limit recovery following stroke. Decreases in numbers of cells expressing CD68 in peri-infarct tissue have also been reported following other interventions that improved function, although these were mostly assessed at later times than in the present study. These interventions have included environmental enrichment [56], inhibition of production of microRNA-155 [57], indomethacin treatment [26, 58], and long-term low-dose treatment with minocycline [26].

Iba1-positive cells in peri-infarct tissue include both resident microglia and tissue macrophages derived from the circulation. However, at 3 days after stroke onset, macrophages are typically only a minor component [17, 18, 59]. In our study, cells expressing CD68 were much less numerous than total Iba1-positive cells and showed a more restricted distribution, with most of the CD68-positive cells localized to within 300 μm of the infarct. The distribution of CD68 expression could be explained if this was induced as a response to signals generated in the infarct or if the immunopositive cells were a separate population predominantly derived from circulating monocytes. The latter possibility seems less likely based on an early investigation demonstrating that CD68 is mainly expressed by a subpopulation of resident microglia at 3 days after photothrombotic stroke [52]. Either of these potential explanations would suggest that the effects of minocycline on peri-infarct microglia are secondary to modifications of cells in the blood or in the infarct. Minocycline can enter brain tissue after intraperitoneal injection but concentrations are much lower than in the blood [60]. Thus, cells in the blood and probably also within the infarct (where there is local breakdown of the blood-brain barrier) will be influenced by higher drug concentrations than resident microglia in the peri-infarct tissue. An important role for blood-borne monocytes in recovery from stroke has been suggested by the finding that functional recovery was greatly reduced when entry of these cells to the damaged brain was blocked [61], although similar effects were not seen following depletion of monocytes/macrophages from the circulation [62].

The limited effects of minocycline on the patterns of Iba1 immunolabeling do not rule out the possibility that the drug treatment had effects on the expression of other proteins in the activated cells. There are few techniques currently available that reliably detect relevant changes in peri-infarct tissue (without contamination from the infarct) and that allow the identification of the cells involved. Recently, there has been considerable interest in possible shifts in microglial properties between an M1 state that is pro-inflammatory and several M2 states that exhibit more anti-inflammatory gene expression profiles [7, 63, 64], although such a classification over-simplifies the responses of microglia in the brain [65, 66]. The few studies examining peri-infarct tissue indicate that genes commonly associated with both the M1 and M2 states are expressed in response to infarction with changes in some studies suggesting greater relative expression of M2-related genes after the first few days [67–72]. However, these investigations have either been limited to the initial 24 h after stroke [67, 68] or only examined individual markers associated with the M1 or M2 state when specifically evaluating the cells within the peri-infarct tissue [69–72]. Furthermore, our assessment of one commonly used M2 marker, arginase-1, in peri-infarct tissue detected substantial expression in cells other than microglia or macrophages (Yew and Sims, unpublished). This observation, which greatly complicates interpretation of studies using arginase-1 as a marker, is consistent with other reports showing expression mainly in neurons in normal brain [73, 74] and upregulation in multiple cell types following stroke [74]. Recently, arginase-1 was detected in blood-derived macrophages in the ischemic core but not in microglia in peri-infarct tissue following permanent middle cerebral artery occlusion [59].

The improved rate of functional recovery induced by the higher-dose treatment with minocycline was associated with a marked increase in the content of the astrocytic cytoskeletal proteins, vimentin, and GFAP, indicating an increase in key features of reactive astrogliosis. Astrocytes exhibit complex responses to infarct formation [12, 16]. The local proliferation of astrocytes and development of a glial scar immediately adjacent to the infarct plays an important protective role in limiting the spread of potentially damaging cells and molecules from the infarct. In addition, reactive peri-infarct astrocytes more distant from the infarct release a range of molecules, including some such as trophic factors and cytokines that can promote neuronal plasticity [12, 16]. Thus, the changes in the responses of peri-infarct astrocytes could have contributed to an improved environment for neuronal plasticity in the peri-infarct tissue.

Minocycline did not significantly alter the marked post-stroke increases in full-length neurocan, a proteoglycan that is also largely generated by astrocytes [45, 47]. Thus, not all components of reactive astrogliosis were apparently equally affected by the treatment. Increases in full-length neurocan form part of changes in perineuronal nets and the extracellular matrix that are thought to promote the formation of new synaptic connections in peri-infarct tissue [16, 75]). Our results suggest that any beneficial effects of this response were not likely to be greatly different between the minocycline-treated rats and those treated with vehicle.

Astrocytes are not usually considered a primary target for the actions of minocycline [28]. Thus, the changes observed in these cells seem more likely to be secondary to effects of the minocycline on other cells. Interestingly, a previous study found that functional recovery was improved by long-term treatment with lower doses of minocycline following photothrombotic stroke and that this was associated with an increase in the number of proliferating astrocytes in peri-infarct tissue [26]. An increased proliferation of peri-infarct astrocytes was also associated with improved functional recovery due to environmental enrichment [76]. As with our findings, these increases in aspects of reactive astrogliosis could have contributed to alterations in the environment in peri-infarct tissue that enhanced neuronal plasticity leading to better recovery.

## Conclusions

Analysis of changes in Iba1 immunoreactivity following photothrombotic stroke confirmed and extended previous reports demonstrating that morphological changes in Iba1-positive cells are detectible in peri-infarct tissue within the first few hours and that marked increases in cell number develop in this tissue over the initial three days. These investigations further revealed that morphological changes indicative of a reactive response spread transiently to other parts of the cerebral cortex and that there were initial losses of Iba1-positive cells in the peri-infarct tissue, which preceded the delayed increases in these cells. The measures of circularity and area fraction of Iba1 immunolabeling provided insights into core features of the reactive responses of microglia and macrophages in the peri-infarct tissue following stroke that are potentially useful in monitoring the effects of treatments on these cell populations.

Treatment with minocycline during the first 2 days after photothrombotic stroke improved aspects of neurological recovery without altering infarct volume. However, this treatment had very limited effects on the stroke-induced changes in the morphology and distribution of Iba1-positive microglia and macrophages in peri-infarct tissue. Although these manifestations of microglial activation have been reported to be modified by minocycline in other studies, they do not seem to be a primary target when treatment with this drug is limited to the early post-stroke period. Substantial reductions in the numbers of CD68-positive cells and increases in features of reactive astrogliosis were seen in peri-infarct tissue following minocycline treatment and could have contributed to the improved outcome.

## Additional file

Additional file 1: **Figure S1.** Additional morphological features of Iba1-positive cells in cortical tissue distant from the infarct at 24 h and 7 days after photothrombotic stroke. **A.** Cell area, **B.** Cell perimeter, **C.** Cell soma area. Results are shown for ipsi02 and contra02 (left panels) and ipsi03 and contra03 (right panels). The ROIs that were analyzed are identified in Fig. 2a. *N* = 3 (7 days) or 4 (24 h); *p < 0.05 compared with the corresponding tissue in the contralateral hemisphere (Student’s t-test). (DOCX 300 kb)

## Abbreviations

Iba1: Ionized calcium binding adaptor molecule 1
GFAP: Glial fibrillary acidic protein
PBS: Phosphate-buffered saline
ROIs: Regions of interest
