# Supplementary figures and images for: Early treatment with minocycline following stroke in rats improves functional recovery and differentially modifies responses of peri-infarct microglia and astrocytes
Source: J Neuroinflammation. 2019 Jan 9;16:6. doi: 10.1186/s12974-018-1379-y (PMC6325745; doi:10.1186/s12974-018-1379-y)

Additional file 1

**A**


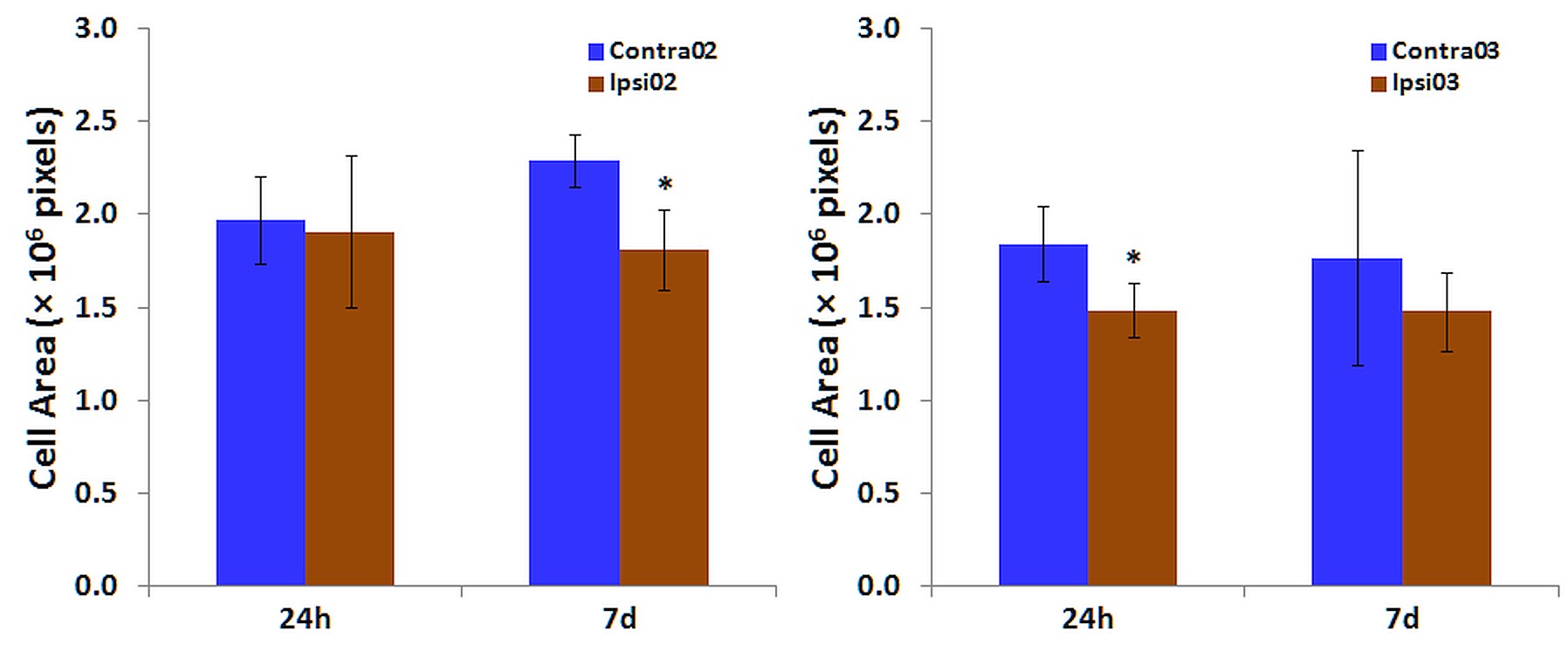


**B**


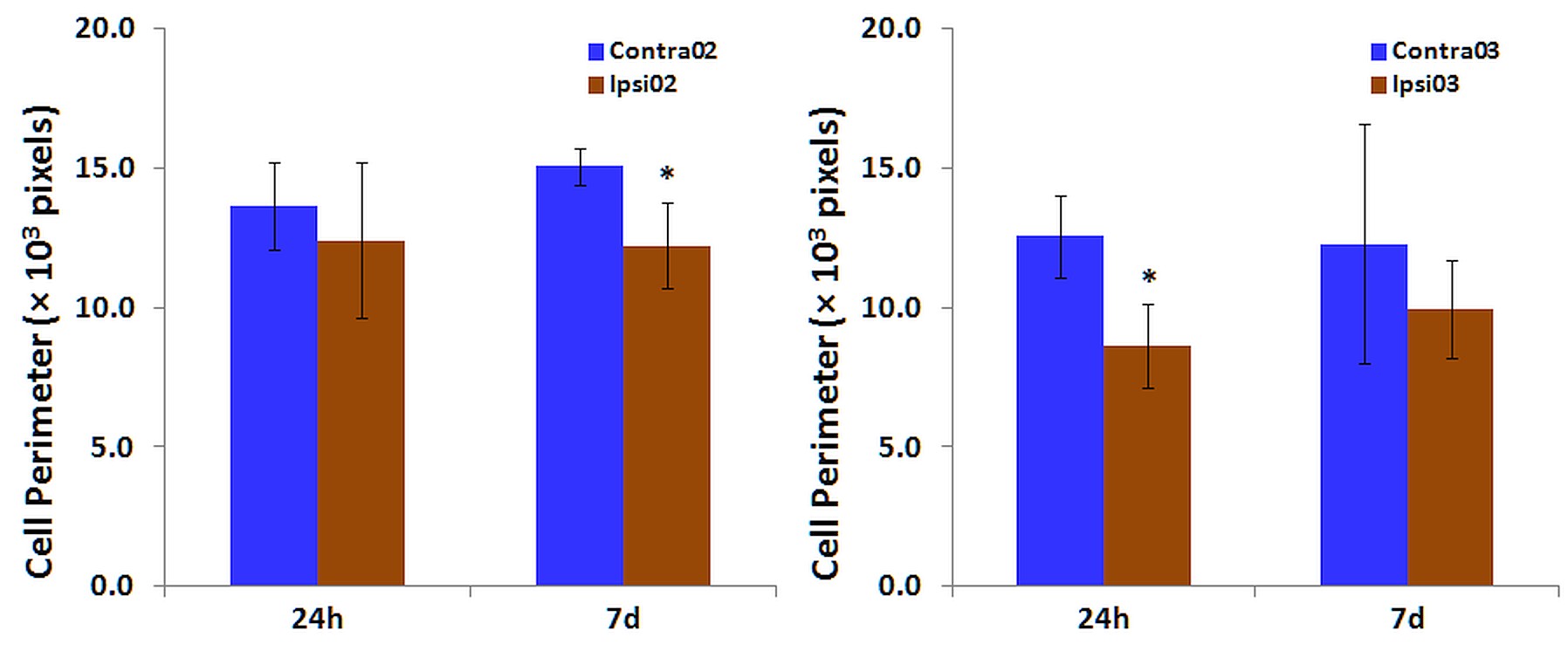


**C**


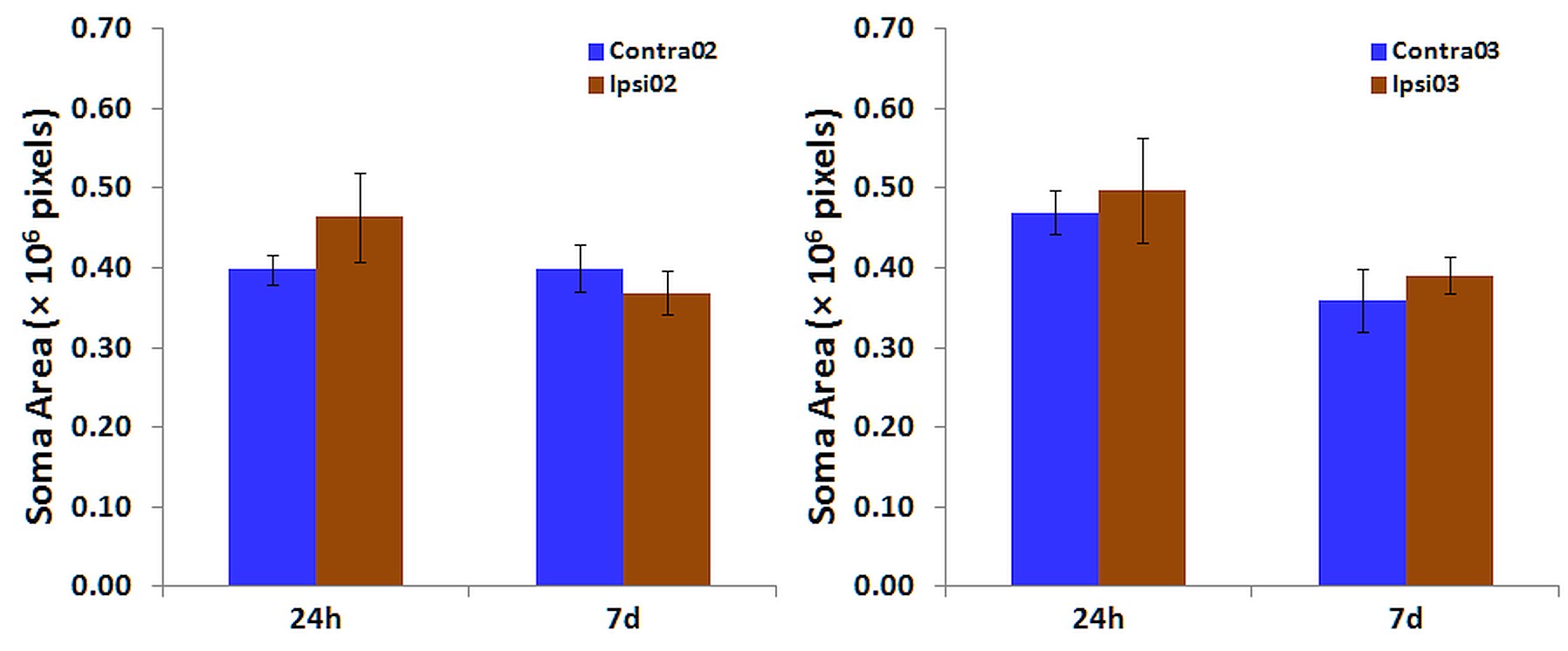

Supplement: Supplementary file 1 — Figure S1. Additional morphological features of Iba1-positive cells in cortical tissue distant from the infarct at 24 h and 7 days after photothrombotic stroke. A. Cell area, B. Cell perimeter, C. Cell soma area. Results are shown for ipsi02 and contra02 (left panels) and ipsi03 and contra03 (right panels). The ROIs that were analyzed are identified in Fig. 2a. N = 3 (7 days) or 4 (24 h); *p < 0.05 compared with the corresponding tissue in the contralateral hemisphere (Student’s t-test). (DOCX 300 kb) [file 12974_2018_1379_MOESM1_ESM.docx]
